# Blocking Epidermal Growth Factor Receptor Signaling in HTR-8/SVneo First Trimester Trophoblast Cells Results in Dephosphorylation of PKB**α**/AKT and Induces Apoptosis

**DOI:** 10.1155/2011/896896

**Published:** 2011-08-21

**Authors:** J. Bolnick, L. Albitar, L. L. Laidler, R. Abdullah, K. K. Leslie

**Affiliations:** ^1^The Reproductive Molecular Biology Laboratory, Division of Maternal-Fetal Medicine, Departments of Obstetrics and Gynecology and Biochemistry and Molecular Biology, The University of New Mexico Health Sciences Center, Albuquerque, NM 87131, USA; ^2^Clinic of Fetal Medicine and Maternal Medicine and Obstetrics and Gynecology, 263 Farmington Avenue Farmington, CT 06030, USA; ^3^Department of Biochemistry and Microbiology, Faculty of Pharmacy, University of Kalamoon, P.O. Box: 222, Deirattiah, Syria; ^4^Division of Gynecologic Oncology, Department of Obstetrics and Gynecology, MSC 105580, 2211 Lomas Boulevard. NE, Albuquerque, NM 87131, USA; ^5^The Center of Reproductive Medicine and Infertility at the Weill Cornell University, CRMI 1305 York Avenue, New York, NY 10021, USA; ^6^Department of Obstetrics and Gynecology, The University of Iowa, Hospitals and Clinics, 200 Hawkins Drive, Iowa City, IA 52242, USA

## Abstract

We identified a major peptide signaling target of EGF/EGFR pathway and explored the consequences of blocking or activating this pathway in the first trimester extravillous trophoblast cells, HTR-8/SVneo. A global analysis of protein phosphorylation was undertaken using novel technology (Kinexus Kinetworks) that utilizes SDS-polyacrylamide minigel electrophoresis and multi-lane immunoblotting to permit specific and semiquantitative detection of multiple phosphoproteins. Forty-seven protein phosphorylation sites were queried, and the results reported based on relative phosphorylation at each site. EGF- and Iressa-(gefitinib, ZD1839, an inhibitor of EGFR) treated HTR-8/SVneo cells were subjected to immunoblotting and flow cytometry to confirm the phosphoprotein screen and to assess the effects of EGF versus Iressa on cell cycle and apoptosis. EGFR mediates the phosphorylation of important signaling proteins, including PKB**α**/AKT. This pathway is likely to be central to EGFR-mediated trophoblast survival. Furthermore, EGF treatment induces proliferation and inhibits apoptosis, while Iressa induces apoptosis.

## 1. Introduction

Epidermal growth factor (EGF) and its receptor (EGFR) are known to play critical roles in cell growth, differentiation, angiogenesis, and inhibition of apoptosis. EGF is a multifunctional 53 amino-acid peptide known for its ability to stimulate proliferation and differentiation in the placenta as well as many other cells and tissues [1]. EGF is important in embryonal implantation and trophoblast differentiation; it is critical for normal placental endocrine function [2]. EGF and its membrane-bound receptor, EGFR, are expressed in the placenta and can be found at all stages of differentiation; however, the signaling events activated by this pathway in the trophoblast are not fully understood. Nevertheless, growth factors such as EGF are believed to be intimately involved with the control of trophoblast physiology, and abnormal levels of EGFR have been reported in the placenta from pregnancies complicated by intrauterine growth restriction (IUGR), preeclampsia, and diabetes mellitus [3]. The association of EGF/EGFR with normal and aberrant placental function makes the study of downstream signaling pathways activated by this growth factor an important area of investigation.

EGFR signals through a series of protein kinases. These are important transducers of information in human cells with the ability to regulate cellular functions by catalyzing the direct phosphorylation of target proteins [4]. Protein kinases operate within complex systems of functionally interconnected signaling protein networks. Identification of the principal EGFR-mediated signaling pathway in trophoblast cells would allow a more complete understanding of the effects of EGF on placental physiology and development. However, to understand EGFR signaling at the global level, it is crucial to track the phosphorylation and the activation of multiple potential protein kinase targets in a reliable, semiquantitative, and accurate manner. In these experiments, we used phosphoprotein profiling with Kinexus technology to identify EGFR targets in HTR-8/SVneo cells. Phosphorylation at 47 sites on 31 potential targets was queried using cells treated with EGF and/or Iressa (gefitinib, ZD1839), a specific blocker of EGFR phosphorylation. Sites were identified for which phosphorylation was induced by EGF and/or inhibited by Iressa. The downstream effects of signaling through the identified pathways were investigated using cell growth and apoptotic assays [5]. 

## 2. Material and Methods

### 2.1. Cell Culture and Treatment

Transformed human trophoblast-8 (HTR-8/SVneo) cells, originally obtained from Professor Charles Graham (Toronto, Canada), were grown to approximately 80% confluence in minimum essential medium (Invitrogen Corporation, Carlsbad, Calif, USA) with 50 mL of fetal bovine serum (Invitrogen, Carlsbad, Calif, USA) [6]. The cells were subsequently serum-starved for 24 h and divided into four groups: control (vehicle alone), EGF (30 ng/mL, Invitrogen, Carlsbad, Calif, USA) for 5 min, Iressa (1 *μ*M, Astra-Zeneca Pharmaceuticals, London, England) for 20 h, and Iressa plus EGF. Iressa was dissolved in DMSO and EGF was dissolved in distilled water according to the manufacturer's protocol. Controls were performed to ensure the absence of effects with the respective vehicles. 

### 2.2. Protein Extraction

Cells were harvested and washed twice with PBS. Protein lysate was obtained according to Kinetworks' guidelines (Kinexus, Victoria, BC). Briefly, protein was extracted using 0.5% Triton X-100 lysis buffer with sonication for 10 seconds on ice to rupture the cells. Cell extracts were then ultra-centrifuged for 30 min at 100,000 ×g. The supernatant was transferred to fresh tubes, and protein concentrations were assayed promptly using Bradford dye and a microplate reader according to the manufacturer's protocol (BioRad, Hercules, Calif, USA). For each sample, 400 *μ*g of protein was transferred to a fresh 1.5 mL Eppendorf screw cap vial and mixed with 4x sample buffer (125 mM Tris-HCl pH 6.8, 4% w/v SDS, 50% v/v glycerol, 0.08% w/v bromophenol blue, and 5%  *β*-mercaptoethanol) in a ratio of 1 : 4 sample to buffer. Samples were then boiled for 4 min, placed in tightly locked tubes and shipped to Kinexus, Inc. for quantification. 

### 2.3. Multipeptide Immunoblotting

Multipeptide immunoblotting was performed according to the manufacturer's instructions using Kinetworks Phospho-Site Screen 1.3 (Kinexus, Victoria, BC), which tracks the abundance of the following phosphoprotein targets: Adducin A (S724), Adducin G (S662), CDK1 (Y15), CREB (S133), ERK1 (T202/Y204), ERK2 (T185/Y187), GSK3-alpha (S21, Y279), GSK3-beta (S9, Y216), JUN(S73), JNK/SAPK (T183/Y185), MEK1/2 (S217/S221), MEK3/6 (S189/S207), MSK1 (S376), NR1 (S896), p38-alpha MAP kinase (T180/Y182), S6K p70 (T389), PKB-alpha/AKT (S473/T308), PKC-alpha (S657), PKC-alpha/beta (T638/641), PKC-delta (T507), PKC-epsilon (S729), PKR (T451), RAF1 (S259), RB1 (S780, S807/811), RSK1 (T359/S365), JNK/SAPK (T183/Y185), SMAD1 (S463/465), SRC (Y418/Y529), STAT1 (Y701), STAT3 (S727), and STAT5 (Y694). By this method, 47 phosphosites on 31 proteins are identified. 

Phosphorylation of each target was assessed using 300 *μ*g of total protein extract resolved on a 13% single-lane SDS-polyacrylamide gel and transferred to a nitrocellulose membrane. Using a 20-lane multiblotter from BioRad (Hercules, Calif, USA), the membrane was incubated with different mixtures of up to three antibodies per lane that react with a distinct subset of phosphorylated cell signaling proteins of known molecular masses, as listed above. After further incubation with a mixture of relevant HRP-conjugated secondary antibodies (Santa Cruz Biotechnology, Santa Cruz, Calif, USA), the blots were developed using ECL Plus reagent (Amersham Pharmacia, Piscataway, NJ, USA), and the signals were quantified using Quantity One software (BioRad) [7]. The relative density of each phosphoprotein band was determined by comparison with the control cells. The results were reported as the percent of binding compared to the control. Peptide densities that varied ±25% or more and were consistently present were considered to be legitimate targets of EGF/EGFR signaling, which could be reliably detected using this method. 

### 2.4. Western Blotting

Confirmation of the multilane immunoblotting data with respect to the principal target identified, PKB*α*/AKT, was performed by standard Western blotting. The antibody for PKB*α*/AKT was specific to the S473 site (Novus, Littleton, Colo, USA). Immunoblotting was also performed for PARP-1, a marker for apoptosis, and for total EGFR (Santa Cruz Biotechnology, Santa Cruz, Calif, USA). HTR-8/SVneo cells were plated in 150 mm dishes and were grown for 24 h and then serum starved. The treatments were control (with DMSO as the vehicle), EGF (30 ng/mL in DMSO) for 5 min, Iressa only (1 *μ*M in DMSO) for 20 h, and Iressa for 20 h + EGF for 5 min. After treatment, cells were harvested and centrifuged for 2 min at 500 ×g. Cell pellets for each treatment were lysed with extraction buffer [1% (v/v) Triton X-100, 10 mM Tris-HCl pH 7.4, 5 mM EDTA, 50 mM NaCl, 50 mM NaF, 20 *μ*g/mL Aprotinin, 1 mM PMSF, and 2 mM Na_3_VO_4_] as previously described by Lange, et al. [8, 9] and subjected to three freeze/thaw cycles. The lysate was cleared by centrifugation at 20,000 ×g for 10 min. The protein concentration of the whole cell lysates was determined by the Bradford method (BioRad, Hercules, Calif, USA). Equal amounts of protein (100 *μ*g) for each treatment were separated by 7.5% SDS-PAGE. Proteins were transferred onto a nitrocellulose membrane (BioScience, Dassel, Germany) and the membrane was then probed with a primary antibody against phospho-AKT (S473), total EGFR, or PARP-1 followed by incubation with an HRP-conjugated secondary antibody. The signal was visualized by chemiluminescence using ECL Western blotting detection reagents (Pierce, Rockford, Il, USA). Actin was used as a loading control. 

### 2.5. Flow Cytometry

 To determine the effects of EGF treatment and/or Iressa blockade on cell cycle and apoptosis, cells were grown to 80% confluence and treated as described above. Cells were then harvested by scraping, washed 3x with PBS, and stained with Krishan's solution (NA citrate 2H_2_O, propidium iodide, 1% NP40, and 10 mg/mL RNAse A). Stained cells were analyzed on a FACS Calibur flow cytometer using CellQuest software version 3.3 (both Becton Dickinson, San Jose, Calif, USA). Data analysis was performed using FlowJo software version 6.4 (Tree Star, Inc., Ashland, Ore, USA). Cells were triggered on forward scatter, gated by forward versus side scatter to exclude debris, and then gated on PI fluorescence area versus width to eliminate doublets. PI area was used to determine the percentage of cells in each compartment of the cell cycle. The proportion of the cells in the sub-G1 compartment was used as a relative indicator for apoptosis. 

### 2.6. Apoptosis Assay

To further confirm the effect of Iressa treatment to block EGF/EGFR signaling on programmed cell death, cells were stained, and the cells undergoing apoptosis were counted. Hoechst 33342 dye (Molecular Probes, Eugene, Ore, USA) was used for these studies, because it provides a rapid fluorescence assay to detect condensed chromatin in apoptotic cells. This blue fluorescent dye stains chromatin at an excitation/emission wavelength of 340–461 nm when bound to DNA. The chromatin of apoptotic cells stains more brightly than the chromatin of normal cells. HTR-8/SVneo cells were seeded in 6-well plates with 3 mL of complete medium added to each well. Cells were grown to 80% confluence in complete media, serum-depleted media, serum-depleted media + EGF (as above), or serum-depleted media + 1 *μ*M Iressa. Twenty hours later, the media was removed and Hoechst dye was added to each well for 30 min. Cells were assessed for apoptotic features using a confocal microscope (Olympus FluoView 1000, Melville, NY, USA). The main morphological characteristics assessed were cellular and nuclear shrinkage (pyknosis), nuclear fragmentation (karyorrhexis), chromatin condensation, blebbing, and the formation of apoptotic bodies [10]. The apoptotic cells were counted in four randomly selected fields by an investigator blinded to the treatment group. The percentage of cells undergoing apoptosis was calculated. 

## 3. Results and Discussion

**Figure 1:**
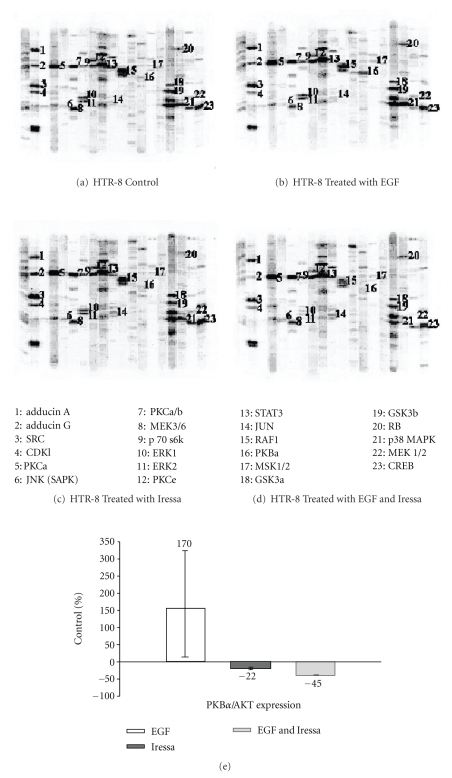
(a) Kinexus multilane immunoblot showing untreated HTR-8/SVneo cells (control). (b) Kinexus multilane immunoblot showing HTR-8/SVneo cells treated for 5 min-with EGF (30 ng/mL). Thirty-one phosphorylated proteins were tracked. Twenty-four proteins (see Table 1) were phosphorylated in HTR-8/SV neo cells. Seven of the proteins were not phosphorylated. PKB*α*/AKT is a principal target which is phosphorylated by EGF (see immunoblot band marked 16). (c) Kinexus multilane immunoblot showing HTR-8/SVneo cells treated for 20 h with Iressa (1 *μ*M) plus EGF (30 ng/mL) for 5 min. Phosphorylation of PKB*α*/AKT was downregulated in the presence of Iressa (immunoblot band marked by 16). (d) Kinexus multilane immunoblot showing HTR-8/SVneo cells treated for 20 h with Iressa (1 *μ*M). Phosphorylation of PKB*α*/AKT is significantly downregulated (see band marked by 16). (e) This graph represents the fold induction or downregulation of pKB*α*/AKT in response to EGF, Iressa, EGF + Iressa normalized to baseline untreated cells.

Using Kinetworks Phospho-Site Screen 1.3, tracking the 47 phosphorylation targets specified in the methods, multiple differences were noted in the phosphoprotein profile of each treatment group using the cutoff of at least 25% difference between treatments. Compared to controls, EGF treated cells demonstrated the upregulation of 12 phosphoproteins [Adducin*α*, CREB, ERK2, Jun, MSK1/2, p38 MAPK, p70 S6K, PKB*α*/AKT (S473 and T308), PKC*α*, RB, SRC and STAT5], whereas five target phosphoproteins [CDK1, NR1, RAF1, SAPK, and MEK3/6] were downregulated. For cells treated with Iressa alone, six phosphoproteins [CREB, GSK3*β*, JUN, PKC*α*, PKC*α*/*β*, and RB] were upregulated and 11 phosphoproteins [Adducin g, CDK1, ERK1, ERK2, MEK1/2, NR1, p38 MAPK, PKB*α*/AKT (S473 and T308), PKCe, RAF1, and SAPK] were downregulated. Representative blots of cells grown under control conditions, compared to cells treated with EGF, Iressa, and EGF + Iressa are shown in Figures 1(a)–1(d). The counts per minute (CPM) for each phosphorylation site under each condition is shown in Table 1.

**Figure 2:**
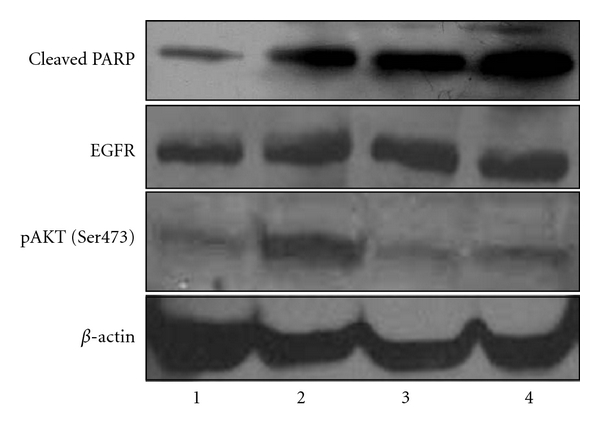
Immunoblot for PKB*α*/AKT, cleaved PARP, and EGFR. HTR-8/SV neo cell treatments are as follows: Lane (1) serum-starved control, (2) with EGF (30 ng/mL, 5 min), (3) Iressa (1 *μ*M, 20 h), and (4) EGF (30 ng/mL, 5 min) + Iressa (1 *μ*M, 20 h).

We reasoned that the principal EGFR phosphorylation target(s) would be both robustly induced by EGF and strongly downregulated by Iressa. Protein Kinase B-*α*/AKT consistently met these criteria, both at S473 and T308. Phosphorylation at S473 is shown in bar graph format in Figure 1(e). Phosphorylation of S473 was induced 328% by EGF compared to control cells and reduced to baseline with the addition of Iressa. Phosphorylation at T308 was also increased over baseline, but the exact percentage could not be calculated, because the baseline phosphorylation at this site in control cells was undetectable. Addition of EGF strongly enhanced phosphorylation at T308, and Iressa inhibited phosphorylation back down to undetectable levels. Confirmation of the Kinexus data was performed by Western blotting and is represented in Figure 2, showing upregulation of phospho-PKB*α*/AKT S473 with EGF treatment but downregulation with Iressa alone or Iressa + EGF. 

Proteins that were phosphorylated in HTR-8/SVneo cells but not significantly affected by EGFR activation or blockade, include the following: Adducin a, Adducin g, CDK1, CREB, ERK1, ERK2, GSK3a, GSK3b, JUN, MEK1/2, MEK3/6, MSK1/2, NR1, PKCa, PKCa/b, PKCe, RAF1, SAPK, SRC, and STAT1 (Table 1). These sites represent pathways that are presumably signaling, but are not under the control of EGFR under the conditions tested. Proteins that were not phosphorylated in HTR-8/SVneo cells include: MEK6, PKCd, PKR, RSK1, SMAD1, SRC, and STAT3. These sites represent downstream signaling targets that are not activated. Hence, these data indicate that of the 47-phosphorylation sites on 31 proteins queried and under the conditions specified, some sites were clearly regulated through EGFR (the major one being PKB*α*/AKT), and some were not. Other sites were phosphorylated but were not influenced by EGFR activation or inhibition. 

**Figure 3:**
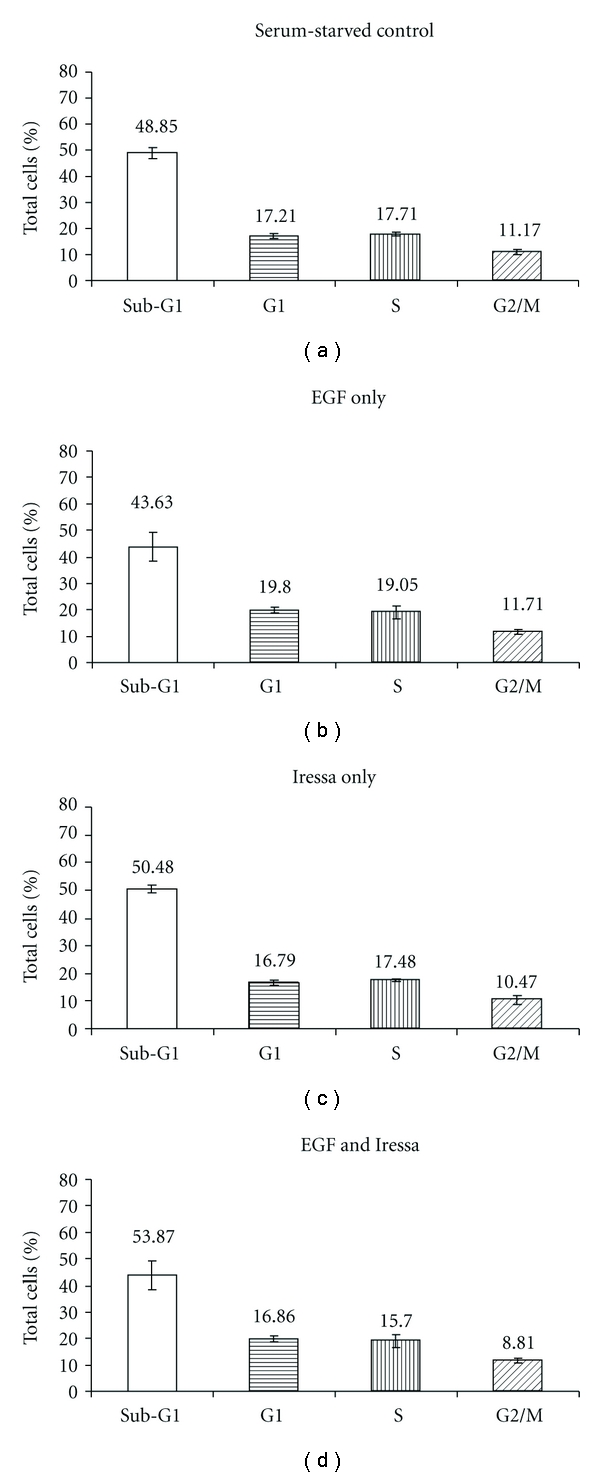
Flow cytometric analysis of cell cycle in response to EGF and Iressa. The sub-G1 fraction consists of a high proportion of apoptotic cells.

Flow cytometry was employed to assess the effects of EGF +/− Iressa and Iressa alone on the cell cycle (Figures 3(a)–3(d)). Compared to control cells grown without serum, EGF treatment induced an increase in the proliferative phases of the cell cycle, G1 and S, by 2.5% and 1.9%, respectively, a meaningful result keeping in mind that over time, this will result in many more cells going through the cycle. On the other hand, Iressa treatment in the presence of EGF reduced the percentage of cells in G1 and S by 3% and 2.8%, respectively, and increased the percent of cells in the sub-G1 phase by 10%. This indicates that Iressa is able to block the proliferative effects of EGF and to enhance cellular apoptosis. Similarly, PARP-1 cleavage, a marker for apoptosis, was induced with Iressa treatment, further confirming the induction of programmed cell death through this pathway. Total EGFR levels were unchanged by EGF and/or Iressa treatment, as shown in Figure 2, indicating that modulation of EGFR (an increase or decrease in expression) is not the reason for altered cell growth and apoptosis in our experiments. 

**Figure 4:**
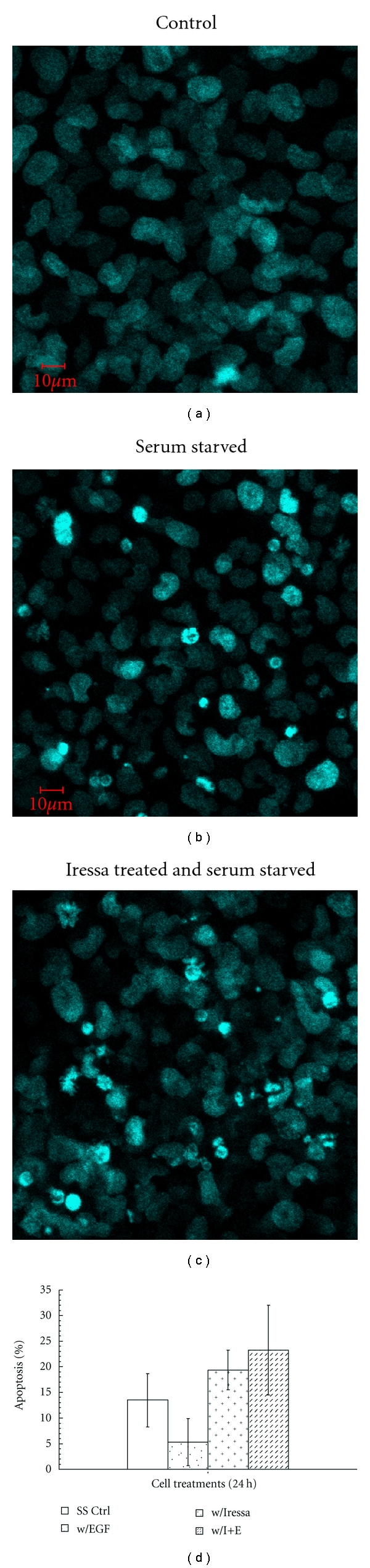
Apoptosis in response to Iressa and serum starvation. HTR-8/SVneo cells as visualized by an Olympus laser confocal microscope at 1000 x. (a) HTR-8/SVneo cells grown in complete media, (b) HTR-8/SVneo cells grown in serum-starved media for 24 h, and (c) HTR-8/SVneo cells were grown in serum-starved media with Iressa (1 *μ*M). Graph (d) represents the average percent of apoptotic cells in complete media (a), serum-starved media (b), Iressa + complete media (picture not shown), and Iressa in serum-depleted media (c). Error bars = standard error of the mean.

The effects of Iressa on apoptosis were studied further using fluorescence microscopy in the presence of Hoechst dye, as shown in Figure 4. Only 2% of cells were apoptotic when grown in complete media with serum. Depletion of serum resulted in an increase in apoptosis to 12%, and the further addition of 1 *μ*M Iressa enhanced cellular apoptosis to 16.5% (Figure 4(d)). Using the Student's *t*-test, significantly more cells were apoptotic in serum-depleted media compared to control conditions in which cells were grown in complete media (*P* < 0.0001). Addition of Iressa to serum-depleted media resulted in a further significant increase in the percent of apoptotic cells (*P* = 0.038). EGF was able to partially inhibit apoptosis compared to serum depletion although this effect did not reach statistical significance in this assay and is thus not shown. The effect of Iressa on cell viability confirmed the apoptotic assays, where treatment resulted in a 31% decrease in HTR-8 cell viability compared to the serum-starved condition (data not shown). 

## 4. Conclusions

The literature strongly supports a primary role for EGFR signaling in the development and the function of the trophoblast [11]. The EGF pathway is central to the control of trophoblast invasion and the inhibition of apoptosis [12, 13]. The purpose of this study was to identify the principal EGFR signaling pathways in first trimester trophoblast cells and to confirm the biological significance of these findings on the induction of programmed cell death. Iressa is known to be a potent oral specific inhibitor of EGFR tyrosine kinase activity and competes for its binding site with adenosine triphosphate on the intracellular domain of the receptor [14]. It was used in this study, along with EGF, to assess which pathways were clearly under EGFR control (induced by EGF and blocked by Iressa) in our cell model.

**Figure 5:**
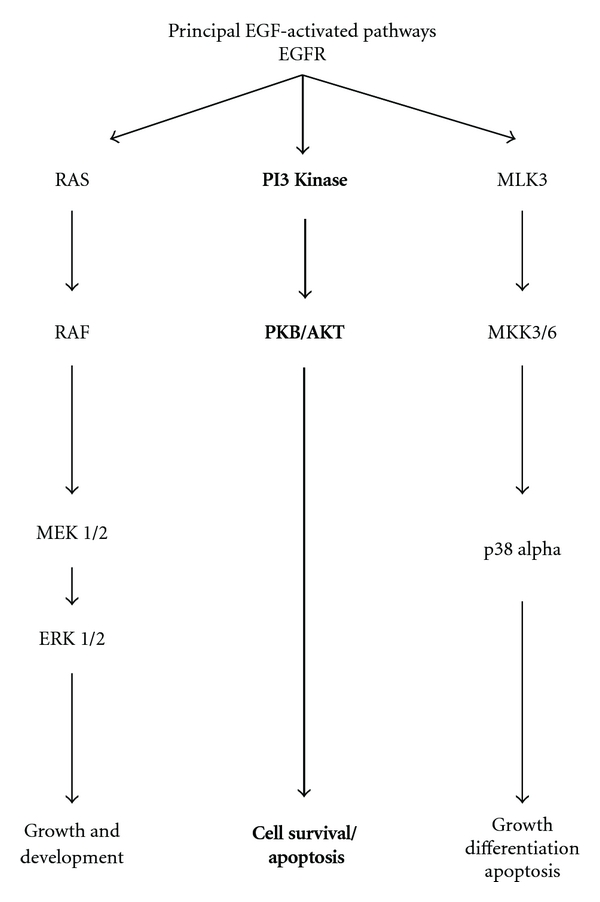
EGF activated pathways in HTR-8sv/neo cells. EGF can signal through multiple pathways and ultimately influence many different downstream peptides. A major effect identified in these studies is through modulation of PKB*α*/AKT (bold).

This study utilized a novel technology to analyze protein expression and phosphorylation changes in 31 proteins. The pathways queried are illustrated in Figure 5. However, this technology has limitations. It is a high-throughput technique but requires confirmation by standard protein assays such as Western blotting, as shown in Figure 2. In addition, the biological significance of the changes in protein phosphorylation should be verified, as we have done in these studies. 

The principal EGFR target reproducibly induced by EGF and inhibited by Iressa was PKB*α*/AKT. Two sites on this protein, S473 and T308, were controlled by EGFR signaling. PKB*α*/AKT phosphorylation occurs through phosphatidylinositol 3 kinases. Full activation of PKB*α*/AKT requires phosphorylation at both S473 and T308. These sites are independently phosphorylated by two intermediates, PI(3,4,5)P3 and PI(3,4)P2, respectively [15]. Interestingly, EGF induced and Iressa blocked both sites, indicating that EGFR signaling results in robust activation of PKB. In response to S473 and T308 phosphorylation, PKB*α*/AKT modulates the phosphorylation of over 40 different downstream peptides. PKB*α*/AKT is involved in the regulation of many biological processes, including cell proliferation, growth, apoptosis, and tumorigenesis, as shown in Figure 5. PKB*α*/AKT is particularly well known as a negative regulator of apoptosis [16]. Phosphorylation of PKB*α*/AKT inhibits JNK activation, leading to the inability to activate the cellular signaling scaffold required to induce apoptosis (17). PKB*α*/AKT has also been implicated in nitric oxide regulation, glucose homeostasis, and insulin signaling [16–18]. Studies have shown that reduction in the phosphorylation of PKB*α*/AKT and the consequences on downstream signaling molecules may have detrimental effects on newborn pregnant mice. Gene disruption of PKB*α*/AKT results in placental insufficiency, fetal growth impairment, and death in the newborn [19].

In summary, these studies indicate that EGF signaling through EGFR activates PKB*α*/AKT as a principal target, and blocking this pathway with the EGFR-specific tyrosine kinase inhibitor, Iressa, results in programmed cell death. We hypothesize that blocking EGFR signaling may result in apoptosis via inhibition of PKB*α*/AKT. Loss or deactivation of PKB*α*/AKT, as suggested by gene disruption models, may be linked to the pathogenesis of IUGR. Further studies are planned to determine whether IUGR in human pregnancies is linked to enhanced trophoblast apoptosis in response to the dephosphorylation of PKB*α*/AKT downstream of EGFR. 

## Figures and Tables

**Table 1:** Kinexus quantitation of phosphosites in response to Iressa and EGF. Table shows counts/minute/ at each phosphorylation site in control cells compared to EGF, EGF + Iressa-treated cells, and Iressa. These data are the quantitative results from the immunoblots in Figure 1.

Phosphoprotein	Control CPM	EGF CPM	Iressa + EGF CPM	Iressa CPM
Adducin a	2525.76	3230.00	2152.00	2589.00
Adducin g	1661.92	1457.00	1969.00	596.00
CDK1	4850.79	3184.00	4417.00	3311.00
CREB	412.29	525.00	1138.00	535.00
ERK1	1620.04	1394.00	1186.00	179.00
ERK2	1051.38	1175.00	913.00	186.00
GSK3a	186.75	189.00	147.00	165.00
GSK3a	3476.29	3194.00	1829.00	3144.00
GSK3b	2330.41	2620.00	2381.00	3279.00
GSK3b				
JUN	289.71	432.00	612.00	801.00
MEK1/2	538.75	522.00	460.00	136.00
MEK3/6	3114.65	2480.00	2341.00	3078.00
MEK6				
MSK1/2				
MSK1/2	284.33	378.00	271.00	337.00
NR1	752.75	351.00	399.00	196.00
p38 MAPK	2201.98	5045.00	2331.00	437.00
p70 S6K		248.00		
p70 S6K	859.82	1218.00	777.00	866.00
**PKBa (S473)**	441.27	1887.00	243.00	326.00
**PKBa (T308)**		415.00		
PKCa	9767.38	12176.00	14306.00	14817.00
PKCa/b	4652.87	5744.00	8439.00	8680.00
PKCd				
PKCe	1533.96	1475.00	1151.00	1382.00
PKR				
RAF1	2223.67	1443.00	1289.00	1307.00
RAF1	5897.68	4372.00	4195.00	3547.00
RB	795.37	1314.00	411.00	1116.00
RSK1				
SAPK	285.45	153.00	179.00	145.00
SMAD1				
SRC				
STAT1	3071.94	3942.00	6013.00	3613.00
STAT3				
STAT5	10297.67	14160.00	11465.00	10810.00
